# Hearing health survey of the population in Bangkok

**DOI:** 10.1186/s12889-024-18424-x

**Published:** 2024-04-12

**Authors:** Suwimol Ruencharoen, Krisna Lertsukprasert, Ravin Suvanich, Jirapat Seesangnom, Mondnath Chockboondee, Wichai Aekplakorn, Chanchai Jariengprasert, Sivaporn Kiatthanabumrung, Tosapohn Wisupagan

**Affiliations:** 1 Department of Communication Disorders, Ramathibodi Hospital, Mahidol University, Bangkok, Thailand; 2 Department of Community Medicine, Ramathibodi Hospital, Mahidol University, Bangkok, Thailand; 3 Department of Otolaryngology, Ramathibodi Hospital, Mahidol University, Bangkok, Thailand

**Keywords:** Hearing loss, Hearing impairment, Community survey, Bangkok

## Abstract

In this cross-sectional random survey among Thai adults living in Bangkok, we aimed to identify the prevalence of hearing problems and examine their relationship with individual factors. We administered a self-report questionnaire and performed pure-tone air conduction threshold audiometry. A total of 2463 participants (1728 female individuals) aged 15–96 years were included. The hearing loss prevalence was 53.02% and increased with age. The prevalence of a moderate or greater degree of hearing impairment was 2.8%. Participants aged 65 years and over had 8.56 and 6.79 times greater hearing loss and hearing impairment than younger participants, respectively. Male participants were twice as likely to have hearing loss and hearing impairment as female individuals. Participants with higher education levels showed less likelihood of having hearing loss and hearing impairment than those with no or a primary school education. Participants who ever worked under conditions with loud noise for > 8 h per day had 1.56 times greater hearing loss than those without such exposure. An inconsistent correlation was found between hearing loss, hearing impairment and noncommunicable diseases (diabetes, hypertension, and obesity). Although most participants had mild hearing loss, appropriate care and monitoring are necessary to prevent further loss in such individuals. The questionnaire-based survey found only people with hearing problems that affect daily communication.

## Background

Hearing is a necessary sense for communication at all ages. Hearing permits humans to learn and progress in speech and language development, which helps lead to a good quality of life. Hearing loss at birth or later in life has multiple effects, including impaired cognitive function, especially in adults and older individuals.Without awareness and a proper diagnosis of hearing loss, including treatment, older individuals may experience dementia earlier than expected [1].

Hearing impairment is one of the most common sensory disorders, according to data from the World Health Organization (WHO), and more than 466 million people worldwide have disabling hearing loss [2]. According to data from the National Institute on Deafness and Other Communication Disorders, 15% of the population over the age of 18 years (37.5 million people) have hearing problems [3]. In a hearing screening study in 2001, Jariengprasert et al. [4] found that the incidence of moderate to severe hearing loss in both ears was 1.7 per 1000 infants born in Ramathibodi Hospital. In 2002, Bunnag et al. [5] performed hearing screening among 980 people between the ages of 60 and 69 years from 14 communities around Siriraj Hospital and found that 52.4% of people had hearing problems.

The prevalence of moderate to severe hearing loss that affects everyday communication varies across the six WHO regions, from 3.1% in the Eastern Mediterranean Region to 7.1% in the Western Pacific Region and 5.5% in the South-East Asia region [6]. Although hearing surveys and studies have been conducted in Thailand, a reliable estimate of hearing loss among the country’s population has not yet been established, hindering the development of policies to manage hearing loss at a national level. On July 4, 2022, the National Health Security Board put forth a resolution to include newborn hearing screening in the Universal Coverage Scheme health benefits package with an announcement that every Thai newborn is eligible for free hearing screening.

The present hearing survey was carried out in the Bangkok area as part of the 6th Thai National Health Examination Survey, conducted as a joint study with the Department of Communication Science and Disorders, Department of Community Medicine, and Department of Otolaryngology of Ramathibodi Hospital, Mahidol University.This preliminary study of hearing health aimed to examine the prevalence of hearing loss and hearing impairment as well as the relationship between hearing problems and individuals factors in the adult population of Bangkok.

## Methods

### Participants

In this study, we used a cross-sectional design and included participants residing in Bangkok who were part of the 6th National Health Examination Survey. The survey method has been described previously [7]. In brief, a multistage sampling method was applied. The first step involved randomly selecting 15 districts from among 50 districts of Bangkok. Second, in each selected district, 3–5 enumeration areas (EUs) were defined in a proportional-to-size approach, resulting in a total of 60 selected EUs. The third step comprised systematic random sampling based on data from the Ministry of the Interior. Using this method, we selected 40 individuals aged 15 years or older from each EU. We aimed to include a total of 2400 participants, accounting for a non-response rate of 20%.

### Methods

The period of data collection was from October 2019 to October 2020. Participants who were unable to complete the questionnaire or communicate effectively with the researcher were excluded from the study. All participants underwent hearing tests using an Amplaid A177 Plus audiometer and TDH39 circumaural headphones to assess the pure-tone air conduction threshold at frequencies of 500–8000 Hz. The examination was conducted in a quiet room with minimal ambient noise, which was periodically measured and controlled to less than 70 dBA [8] and adjusted via biological calibration to ensure accurate measurement of hearing ability. Audiometric measurements were conducted by trained audiologists under the supervision of senior audiologists (SR, KL, RS, JS, MC) from the Department of Communication Disorders.

Pure-tone thresholds were obtained using short tone bursts presented at a clearly audible level, usually 35–50 dBHL (relative to high ambient noise), then decreasing by 10-dBHL intervals until the patient no longer responded, and increasing again in 5-dB steps. To determine the hearing threshold, two out of three responses in ascending order at the same intensity (dBHL) were required. Thresholds at 500, 1000, 2000, 4000 and 8000 Hz for each ear were recorded, and the thresholds at 500–4000 Hz for each ear were averaged (pure-tone threshold average, PTA) and analyzed. Participants with hearing thresholds higher than 25 dBHL at any frequency were identified as having hearing loss. The hearing impairment grading system was applied to indicate the initial hearing levels (PTA) based on the better ear and the level of hearing ability that has an impact on communication (Table 1) [9].

**Table 1:** Definition and characteristics on grades of hearing impairment according to level of hearing loss, listening performance and recommendation [9]

Grades of Hearing Impairment	Corresponding Audiometric ISO Value (dBHL)	Listening Performance	Recommendations
0: no impairment	≤ 25 both ears	No or very slight hearing problems. Able to hear whispers	None or hearing conservative care
1: slight impairment	26–40 (better ear or both ears)	Able to hear and repeat words spoken in normal voice at 1 m	Counseling May need Hearing aids
2: moderate impairment	41–60 (better ear or both ears)	Able to hear and repeat words using raised voice at 1 m	Usually recommend for Hearing aids
3: severe impairment	61–80 (better ear or both ears)	Able to hear some words when shouted into better ear	Hearing aids needed. If no hearing aids available, lip-reading should be taught
4: profound impairment (deafness)	≥ 81 Both ears	Unable to hear and understand even a shouted voice	Hearing aids may help in understanding words. Additional rehabilitation needed. Lip-reading and sometimes signing essential

dBHL: decibel hearing level; ISO: International Organization for Standardization; m: meter

Participants with hearing loss were referred to an otolaryngologist for further analysis of their background and for otologic examination, counseling, and/or consultation with their local service unit, according to the patients’ rights, for further care and monitoring.

### Self-report questionnaire on individual factors

All participants in this study were drawn from the 6th National Health Examination Survey and asked to complete the questionnaire and complete all tasks and tests within 3 h. The basic information of all participants was retrieved from the main survey project. In the hearing assessment portion, a short form yes/no self-report questionnaire was administered to collect information on respondents’ hearing-related behaviors, including their occupation, whether they worked in a noisy environment for more than 8 h per day, whether they listened to music on a mobile phone for more than 4 h per day, and whether they experienced tinnitus.

### Statistical analysis

Descriptive statistics were used to determine the frequency and percentage of study variables. The relationship between hearing level and individual factors was analyzed using logistic regression. Because the population had a wide age distribution, the results were calculated as age-adjusted odds ratios with 95% confidence intervals. The significance level was set to 0.05. The estimates were calculated by incorporating probability weights derived from the complex survey design and presented in percentages of the population [7]. The chi-square (*X*^*2*^) test was used to compare the percentage of hearing loss between subgroups of individual parameters, with a significance level of 0.05. All data were analyzed using SPSS version 18 (SPSS Inc., Chicago, IL, USA).

## Results

A total of 2463 participants were included, with an average age of 56.96 ± 15.11 years (range, 15–96 years). The weighted average ambient noise was 63 ± 7.44 dBA, an acceptable level according to ASHA guidelines for identifying hearing screening failures with a gradual increase in ambient noise up to 70 dBA [8].

According to hearing threshold, all participants were divided into two categories, those with normal hearing and those with hearing loss. The two groups were classified into subgroups according to their individual characteristics, as shown in Table 2. The proportions in each subgroup with normal hearing and hearing loss were significantly different (*p* < 0.001). The proportion of male participants with hearing loss was greater than that among female participants (66.12% vs. 47.45%). The proportion with hearing loss in participants aged 65 years or more was greater than that in younger participants (84.89% vs. 37.30%). The proportion with hearing loss among participants with a primary school education and no education was larger than that among participants with a secondary school and those with higher education levels (63.82% vs. 43.68% and 40.74%, respectively). The proportion of participants with hearing loss was highest among widows and widowers compared to divorced, single and married (70.49% vs. 61.06%, 31.40%, 53.41%). The rate of hearing loss among participants with diabetes was higher than that in participants without diabetes (80.63% vs. 49.55); the same was true for participants with hypertension in comparison with participants who did not have hypertension (67.13% vs. 47.46%). The proportion with hearing loss among participants with tinnitus was greater than that among participants without tinnitus (66.92% vs. 49.43%). Although participants with obesity had a higher rate of hearing loss, this was not statistically significant (54.63% vs. 48.83%). Overall, hearing loss was found in 1306 of 2463 participants, with a hearing loss prevalence of 53.02%.

**Table 2:** Percentage of hearing loss according to personal profiles and physical characteristics

Personal data		Normal hearing n (%)	Hearing loss n (%)	Total n (%)	${\chi }^{2}$	*P*-Value
Gender	Male Female	249 (33.88) 908 (52.55)	486 (66.12) 820 (47.45)	735 (29.84) 1,728 (70.26)	72.15	< 0.001
Age (year)	< 65+ $\ge$65	1,034 (62.70) 123 (15.11)	615 (37.30) 691 (84.89)	1,649 (66.95) 814 (33.05)	495.61	< 0.001
Education	None/ Primary School	402 (36.18)	709 (63.82)	1,111 (46.92)	108.69	< 0.001
	Secondary Vocational School/College/Post graduate	419 (56.32)	325 (43.68)	744 (31.42)		
	Higher Vocational School/College/Post graduate	304 (59.26)	209 (40.74)	513 (21.66)		
Marriage status	Married Single Divorced Widow/Widower	636 (46.59) 295 (68.60) 81 (38.94) 103 (29.51)	729 (53.41) 135 (31.40) 127 (61.06) 246 (70.49)	1,365 (58.04) 430 (18.28) 208 (8.84) 349 (14.84)	128.65	< 0.001
Diabetes Mellitus	Absent Present	1,027 (50.44) 104 (19.37)	1,009 (49.55) 433 (80.63)	2,036 (79.13) 537 (20.87)	166.56	< 0.001
Hypertension	Absent Present	919 (51.17) 212 (32.87)	830 (47.46) 433 (67.13)	1,749 (73.06) 645 (26.94)	73.19	< 0.001
Obesity (BMI$$ \ge $$ 25 kg/$$ {m}^{2}$$)	Absent Present	481 (51.17) 637 (45.37)	459 (48.83) 767 (54.63)	940 (40.10) 1,404 (59.90)	1.1408	0.285
Tinnitus	Absent Present	981 (50.57) 171 (33.08)	959 (49.43) 346 (66.92)	1940 (78.96) 517 (21.04)	50.15	< 0.001
Total		1,157 (46.98)	1,306 (53.02)	2,463 (100)		

Of 1306 participants, 1264 were referred for an otologic examination. After the examination, 275 patients (21.76%) showed abnormal findings, with 99 patients (7.83%) having problems in both ears and 176 showing abnormalities in one ear (13.92%). The following outer and middle ear abnormalities were identified in these patients: (1) impact cerumen (*n* = 129, 10.21%); (2) tympanic membrane (TM) perforation (*n* = 36, 2.85%); (3) eardrum abnormality (*n* = 35, 2.77%); (4) other abnormalities including old healed TM perforation, retracted TM, erythematous TM, tympanosclerosis, otitis externa, and otomycosis (*n* = 49, 3.87%); and (5) various findings in both ears such as minimal cerumen, partially visible TM, foreign body and etc. (*n* = 26, 2.06%).

Table 3 shows cumulative proportions with hearing loss among 1306 participants, categorized by age interval and sex. The prevalence of hearing loss increased with advancing age intervals of 15, 25, 35, 45, 55, 65 and 75 years, with rates of 1.67%, 7.84%, 17.76%, 31.06%, 57.12%, 79.96%, and 96.33%, respectively. Most patients with hearing loss were aged 55 years or over, accounting for 1108 patients (44.98%). In the age group less than 55 years of age, 198 (8.04%) patients were identified as having hearing loss. Specifically, 691 (28.05%) patients aged 65 years or older exhibited hearing loss. With regard to sex, 486 (66.12%) of 735 men and 820 (47.45%) of 1728 women had hearing loss.

**Table 3:** The cumulative percentage of participants with hearing loss according to age interval and gender. Participants with hearing loss at any frequency of all degree were included. Cumulative percentage shows the prevalence of hearing loss advancing with age which the majority of cases were found in the participants aged 55 years and older

Age (years)	Hearing Loss			
	Male	Female	Total	
	n (%)	n (%)	n (%)	Cumm n (%)
15–24 *n* = 120	1 (1.75)	1 (1.59)	2 (1.67)	198 (8.02)
25–34 *n* = 102	4 (9.37)	4 (5.80)	8 (7.84)	
35–44 *n* = 214	23 (34.32)	15 (10.20)	38 (17.76)	
45–54 *n* = 483	63 (55.26)	87 (23.58)	150 (31.06)	
55–64 *n* = 730	149 (74.5)	268 (50.57)	417 (57.12)	1108 (44.98)
65–74 *n* = 569	156 (90.17)	299 (75.51)	455 (79.96)	
> 75 *n* = 245	90 (98.90)	146 (94.81)	236 (96.33)	
Total *n* = 2463	486 (66.12)	820 (47.45)	1306 (53.02)	

Cumm = cumulative percentage

Table 4 shows participants categorized according to degree of hearing impairment, age, and sex. According to hearing impairment classifications (Table 1), 659 participants (26.76%) had hearing loss at some frequencies with a normal PTA level (≤ 25 dBHL); 295 participants (11.98%) with hearing loss at a high frequency (8000 Hz) were classified having no hearing impairment. A mild degree of hearing impairment was found in 283 participants (11.98%). Sixty-nine of 2463 participants (2.79%) were found to have moderate to profound degrees of hearing. A severe degree of hearing impairment was most commonly observed in the age group 45 years and over. Among those aged 75 years or more, 34 participants (1.38%) had a severe degree of hearing impairment, followed by 21 participants (0.85%) in the age group 65–74 years. Moderate to severe and profound hearing impairment was identified in 37 women (1.50%) and 32 men (1.30%). Overall, the prevalence of all levels of hearing impairment was 14.29% (352/2463), including 2.8% (69/2463) with a moderate to severe or profound degree of impact on everyday communication.

**Table 4:** Percentage of participants with degrees of hearing impairment according to age interval. (With an average hearing degree in the good ear)

		Degrees of Hearing Impairment						
Para- meters	Normal Hearing	No HI		Mild HI	Moderate HI	Severe HI	Profound HI (deaf)	Total
		HL with normal PTA	HL at 8000 Hz					
	n (%)	n (%)	n (%)	n (%)	n (%)	n (%)	n (%)	n (%)
Age (years)								
15–24	118 (98.33)	2 (1.67)						120 (4.87)
25–34	94 (92.16)	4 (3.92)		4 (3.92)				102 (4.15)
35–44	176 (82.24)	16 (7.48)	18 (8.41)	4 (1.87)				214 (8.69)
45–54	333 (68.94)	95 (19.67)	39 (8.07)	14 (2.90)		2 (0.41)		483 (19.61)
55–64	313 (42.88)	222 (30.41)	113 (15.48)	70 (9.59)	10 (1.37)	2 (0.27)		730 (29.64)
65–74	114 (20.04)	231 (40.60)	103 (18.10)	100 (17.57)	18 (3.16)	3 (0.53)		569 (23.1)
> 75	9 (3.67)	89 (36.33)	22 (8.98)	91 (37.14)	29 (11.84)	3 (1.22)	2 (0.82)	245 (9.94)
Gender								
Male	249 (33.88)	263 (35.78)	74 (10.07)	117 (15.92)	27 (3.67)	3 (0.41)	2 (0.27)	735 (**29.84**)
Female	908 (52.55)	396 (22.92)	221 (12.79)	166 (9.60)	30 (1.73)	7 (0.41)		1728 (**70.26**)
Total	1,157 (46.98)	659 (26.76)	295 (11.98)	283 (11.49)	57 (2.31)	10 (0.41)	2 (0.08)	2463 (100)

PTA = pure tone average, dBHL = decibel-hearing level, HL = hearing loss, HI = hearing impairment, F = frequency

Table 5 presents the correlation between hearing loss or hearing impairment and individual factors in terms of age, sex, common non-communicable diseases (NCDs), and hearing-related behaviors. Compared with younger groups, participants aged 65 years and over had more significant hearing loss and hearing impairment, by 8.56 and 6.79 times, respectively. More male than the female participants had hearing loss and hearing impairment, by 2.56 and 2.35 times, respectively. Participants with a secondary education and no or primary school education were found to have 1.36 and 2.66 times greater hearing loss and 1.58 and 2.69 times greater hearing impairment in comparison with participants who had a higher education level.

**Table 5:** Age adjusted Odds Ratio (OR) for personal factors correlation with Hearing Loss (HL) and Hearing Impairment (HI)

Personal Factors		Odds Ratio (95% CI) (HL)	Odds Ratio (95% CI) (HI)
Age (years)	< 60 ≥ 65	1** 8.56 (6.51–11.24)	1** 6.79 (4.72–9.77)
Gender	Female Male	1** 2.56 (1.94–3.38)	1** 2.35 (1.66–3.33)
Education	-Higher vocational school/college/post-graduate -Secondary school/ vocational school -No/ primary school	1** 1.36 (0.95–1.96) 2.66 (1.87–3.78)	1** 1.58 (0.84–2.95) 2.69 (1.54–4.70)
Diabetes Mellitus	No Yes	1 1.36 (0.93–1.97)	1 0.95 (0.59–1.52)
Hypertension	No Yes	1** 1.75 (1.27–2.42)	1 1.55 (1.05–2.29)
Obesity	No Yes	1 0.97 (0.74–1.27)	1** 0.89 (0.60–1.30)
Working in a noisy environment ≥ 8 h.	No Yes	1** 1.56 (1.13–2.17)	1 1.90 (1.21-3.00)
Listening to Personal recreating device	< 1 h 1–4 h > 4 h	1 0.75 (0.53–1.07) 0.59 (0.37–0.94)	1** 0.71 (0.42–1.19) 0.61 (0.33–1.33)

CI-confidence interval, *p -value < 0.05, ** *p* -value < 0.001

We found that participants with hypertension had 1.75 times greater hearing loss than those without this condition, with no significant correlation to hearing impairment. Participants with obesity were found to have 0.89 times lower hearing impairment than those without obesity, with no significant correlation to hearing loss. No correlation was found between hearing problems and diabetes. Those with exposure to loud noise at work ≥ 8 h per day were found to have greater hearing loss by 1.56 times in comparison participants who had no such exposure, with no correlation found with hearing impairment. However, a significant negative correlation was found with hearing impairment among participants who listened to music on a mobile phone for < 1 h per day compared with those who listened to music on their phone for 1–4 and > 4 h, by 0.71 and 0.61 times, respectively.

## Discussion

In this research, we explored the prevalence of hearing loss and hearing impairment among the Thai population residing in Bangkok and the association of hearing problems with related individual factors. According to our results, the prevalence of all degrees of hearing loss was as high as 53.02% of the total participants. Most participants with hearing loss (44.98%) were in the age group 55 years or over, consistent with a study by Newall et al. (2020) [10]. The reported prevalence of hearing loss in the Philippines is 48.1%, and the prevalence of hearing loss in adults’ ranges from 34.1 to 39.4% [10–12]. Bunnag et al. (2002), surveyed a population between age 60 and 69 years and reported a hearing loss prevalence of up to 52.4% [5]. When considering the impact of age, it has been observed that the prevalence of hearing loss increases with advancing age, which is consistent with the findings of Xuewen et al. (2021) [13].

Using the hearing impairment grading system, the prevalence of moderate to severe hearing loss among our population in Bangkok was 2.8%, consistent with the findings of Ferrite [14] in northwest Cameroon, where the prevalence of moderate or greater hearing loss was reported to be 3.6%. However, this prevalence was lower compared with studies in the Philippines and China, with a reported prevalence of 15% and 16.3%, respectively [10, 15] Differences in the prevalence of hearing loss/hearing impairment are probably owing to factors such as differences in population size, sampling strategies, definitions or criteria for grading hearing loss, and the prevalence of outer and middle ear diseases in the study population.

In Southeast Asia, the prevalence of moderate to severe hearing loss affecting everyday communication is 5.5%, derived from responses to a questionnaire [6]. Reports from a large-scale Thai survey among 87,134 adults aged 15–87 years revealed that 8.5% had hearing problems, 0.13% had profound hearing loss, 7.7% had hearing problems from the age of 13 years, and most respondents aged 50 years and older experienced hearing problems [16]. A hearing loss survey with one-answer questions (sensitivity 78%, specificity 67%) showed an increase in comparison with test results for minor hearing loss, with no relation to sex or age [17]. Moreover, results of the 6th National Health Examination Survey, the leading nationwide project using questionnaires in hearing assessment, revealed that 1.2% of elderly respondents had severe hearing problems [7]. A questionnaire and interview about hearing problems and hearing ability including ‘yes/no’ questions revealed a lower prevalence than instrumental measurement of hearing function.

The results of our study showed that most participants with hearing loss (approximately 50%) had no hearing impairment and no impact on their daily communication. This type of hearing loss occurs silently, progresses slowly, and is difficult to detect, resulting in the lack of any prevention measures implemented to slow progression of the condition. In contrast, hearing loss in both ears or in only one ear, at the speech frequencies required for daily communication, is detected more easily, enabling affected individuals to seek timely and appropriate care. Our results showed that hearing loss increases with age [18] and affects daily activities. For those aged 65 years or over, the condition may be associated with a greater risk of dementia.

### Relationship between hearing loss/hearing impairment and individual factors

#### Age

All the human senses decline with age; however, hearing function is most commonly affected [19]. Age has a strong impact on hearing loss, which is consistent with the results of several studies [20–30]. At age 65 years or over, we found a significant 8.56 times greater likelihood of experiencing hearing loss as compared with younger ages.

#### Sex

In this study, we found that men were twice as likely to develop hearing loss as women. A Korean study reported significantly higher rates of hearing loss at 4000 Hz and 8000 Hz among men than in women [31]. Wang revealed a prevalence of hearing loss of 37.6% in men and 36.0% in women [32].

There are variations in the results of different related studies, including the leading projects nationwide, with a prevalence of hearing problems among men and women of 41.2% and 54.9%, respectively [7], reported in one study. Another study found an average prevalence of hearing loss for women and men of 42.3% and 36.8%, respectively [13]. These differences may be owing to different survey methods and lifestyles in different countries.

#### Education level

The results of our study showed that fewer participants with higher levels of vocational and postgraduate education had hearing loss and hearing impairment in comparison with participants who had a secondary or elementary school education or no education. This is consistent with the results from a study in China on lifestyle and environmental factors [13] and is probably owing to better health literacy with a higher education level.

Several studies have revealed a relationship between hearing loss and NCDs [33–35], in contrast to our observation regarding the association of hearing impairment with diabetes, hypertension, and obesity. In this study, hypertension was correlated with hearing loss but diabetes and obesity were not. Additional disease-related indicators such as blood pressure level, blood glucose level, and changes in body mass index should be evaluated to identify whether any correlation exists with hearing loss. Confounding factors such as smoking, sleep apnea, dyslipidemia, and other NCDs not considered in this study, which exists in some participants would result in a high prevalence of hearing problems [7]. Whether these NCDs contribute to hearing loss should be investigated in further research.

Noise-induced hearing loss is related to several factors including the type of sound, loudness, duration of exposure, total cumulative lifetime dosage, and individual susceptibility [36, 37]. In this study, exposure to noise in the working environment for a long period showed a higher correlation with hearing loss whereas listening to other types of sound, such as listening to music using a mobile phone, showed a reverse correlation. The information from our interviews did not include all factors affecting hearing ability, so more comprehensive interviews and questionnaires [38] should be conducted for more detailed investigation.

## Conclusion

The results of this research showed that our participants from the general population of Bangkok aged 15 years and over had a prevalence of hearing loss of 53.02%, which increased with age. The majority of the participants (50.23%), had no or mild hearing impairment giving a prevalence of *hearing impairment* of 2.8%. Individuals who are asymptomatic or had mild symptoms require appropriate care and monitoring to prevent premature hearing loss. We also found that participants aged between 55 and 74 years had moderate levels of hearing loss, up to profound hearing loss. Further diagnosis and hearing aids must be provided to such individuals to improve their communication and quality of life.

Older people who have noise exposure, symptoms of tinnitus, notification by a close relative about hearing problems, or underlying diseases related to hearing loss should seek hearing evaluation, proper assessment, and medical care. Our questionnaire-based survey could only identified people with hearing problems that affect their daily communication, which is not equivalent to the actual prevalence of hearing loss. The results of this research provide baseline information for further population surveys in the region to improve hearing-related policies at the national level and budgeting for the provision of hearing aids for individuals with hearing disorders.

### Limitations

This study was conducted in Bangkok, so the estimated prevalence cannot be generalized to the national level. During the COVID-19 outbreak, there were limitations in conducting data surveys and managing research teams while adhering to COVID-19 prevention measures. These limitations included participants’ refusal to be tested and challenges in communication owing to the use of facial masks. In future surveys, the use of technologies such as mobile applications should be considered to enhance self-screening for hearing impairment. Ongoing studies should explore the effectiveness of such applications.

According to the hearing loss scale related to hearing experience of the World Report on Hearing 2021, normal hearing should remain at a hearing threshold of less than 20 dBHL [6]. If applied in this study, the scale would reduce the percentage of participants with normal hearing from 46.98 to 2.19%, resulting in an extremely high prevalence of hearing loss (i.e., 98%). However, this scale seems appropriate for pediatric studies conducted in a well-controlled measurement environment, such as a sound-proof room. In the present hearing survey, most measurements were conducted in a partially controlled environment, usually with relatively high ambient noise.

## Data Availability

The datasets generated and/or analyzed in the current study are not publicly available in English, only in Thai language. However, these data are available from the corresponding author on reasonable request. The data that support the findings of this study are available from Mr. Weerapat Punkla (pw.golfzawe@gmail.com); however, restrictions apply regarding the availability of these data, which were used under license for the current study.
